# Decolonizing healthcare: the role of traditional medicine in building inclusive health systems for Cameroonian women

**DOI:** 10.1186/s12906-025-05133-0

**Published:** 2025-10-17

**Authors:** Emmanuel Aoudi Chance, Papa Théophile, Louise Renee Loe

**Affiliations:** 1 VID Specialized University, Ulriksdal 10, Bergen, Norway; 2 Hôpital Protestant de Ngaoundéré, Ngaoundéré, Cameroon; 3 Yaoundé, Cameroon

**Keywords:** Traditional medicine, Decolonizing healthcare, Cameroon women’s health, Health equity, Gender and indigenous knowledge

## Abstract

**Background:**

Despite progress in expanding formal healthcare services, large segments of the population in Cameroon—particularly women in rural and peri-urban communities—continue to rely on traditional medicine for their primary health needs. This research explores the intersection between gender, indigenous healing practices, and health economics, framed within the global movement to decolonize health systems. Specifically, this study investigates the socio-economic roles of traditional medicine for women in northwest, center, and southwest Cameroon, the systemic barriers they face in integrating their practices into formal healthcare, and how their experiences can inform the development of inclusive, culturally grounded, and sustainable health systems in Cameroon.

**Methods:**

This qualitative study, employing a decolonial-feminist lens and critical theory, explores the potential of traditional medicine, as experienced and practiced by women in Cameroon, to contribute to more inclusive and decolonized health systems. Data was collected through participant observations and in-depth interviews with women traditional healers and users. A total of 30 respondents. Challenging the dominance of Western biomedicine, the research centers indigenous knowledge and women’s agency as healers and knowledge custodians. By valuing lived experiences and contextual understanding, this approach aims to identify pathways for equitable integration of women-led traditional healing practices into formal healthcare, fostering health system decolonization and gender justice.

**Results:**

Traditional medicine plays a crucial economic and psychosocial role for women—as women-led caregivers and community health entrepreneurs, often providing vital income and social capital in resource-limited settings. However, legal exclusion, epistemic bias (undervaluing indigenous knowledge), and lack of integration into the national health policy create systemic barriers. Participants also expressed that traditional practices are not only therapeutic but symbolic of cultural identity, safety, and autonomy in decision-making—especially in maternal and reproductive health.

**Conclusion:**

Integrating traditional medicine—through policy reform, education, and economic support—could significantly contribute to inclusive healthcare delivery in Cameroon, potentially improving healthcare access for marginalized women and fostering culturally relevant and sustainable health solutions. Specifically, our findings suggest the need for policies that facilitate formal recognition of women traditional healers and the establishment of collaborative frameworks between traditional and biomedical practitioners. This research highlights the need for policy shifts that recognize the value of women’s indigenous knowledge and facilitate their meaningful inclusion in national health systems.

**Supplementary Information:**

The online version contains supplementary material available at 10.1186/s12906-025-05133-0.

## Introduction

In Cameroon, as in much of Sub-Saharan Africa, health systems are shaped by the legacies of colonialism, the dominance of Western biomedicine, and the enduring presence of indigenous knowledge traditions [1]. Within these contexts, women have historically played central roles, serving as caregivers, herbalists, midwives, birth attendants, and spiritual healers [2]. As a result, traditional medicine remains a primary source of care for a large segment of the population, where traditional healers are often the first—and sometimes only—point of contact for healthcare, especially in managing chronic illnesses, maternal and reproductive health, and mental health concerns [3, 4]. At the global level, the WHO Traditional Medicine Strategy (2014–2023) supports the integration of indigenous systems into national frameworks, particularly in low- and middle-income countries [5]. This resilience of traditional medicine, offering not only accessible alternatives to formal healthcare but also culturally grounded care embedded in social, spiritual, and ecological contexts, is a source of inspiration and hope for marginalized and vulnerable Populations and individuals seeking holistic and personalized care [2, 6]. Accessible and culturally grounded care fosters inspiration and hope by treating the whole person—mind, body, and spirit—within their cultural context. This approach builds trust and understanding, making individuals feel seen and validated. It empowers them by giving them a sense of control over their own healing journey and strengthens their connection to their identity, land, and spiritual well-being, offering a more complete and hopeful path to health [7–9]. The deep cultural integration of traditional practices, and the subsequent popular demand for formal recognition, is a cornerstone of the argument for decolonizing healthcare. This is powerfully illustrated by a 2022 survey from the Centre and Southwest regions. It found not only that 89.3% of adults had consumed herbal teas—a practice often managed and transmitted by women—but, critically, that 90.1% supported their official prescription in hospitals. This overwhelming public support demonstrates a clear mandate from the community to build a truly inclusive health system by legitimizing and integrating trusted, traditional remedies [10].

Asonganyi (2013) describes the relationship between conventional and traditional medicine in Cameroon as complex, with ongoing use of traditional practices amid mistrust and limited collaboration between the systems [11]. Despite this widespread reliance, traditional medicine remains undervalued in national health policy frameworks, raising urgent questions about inclusivity, equity, and epistemic justice.

The urgency of addressing these barriers highlights the critical need for systemic reform within the healthcare framework and motivated action on dismantling the colonial-era policies and epistemic biases that continue to undervalue traditional medicine. Biomedical systems, rooted in colonial histories and reinforced through post-independence reforms, have largely ignored or suppressed indigenous approaches to health and well-being. These holistic approaches stand in contrast to the biomedical model by viewing health not merely as the absence of disease, but as a balance of an individual’s physical, spiritual, and emotional well-being in connection with their community and their land. By fostering cultural identity, social cohesion, and addressing the root causes of illness, these systems provide a powerful framework for promoting positive health outcomes [12–14]. This has resulted in a health system disconnected from the lived reality of those it claims to serve—a system where care is often geographically inaccessible, financially prohibitive, delivered in a language patients may not fully understand, and founded on a clinical model that dismisses the very cultural and spiritual beliefs central to their lives. Decolonizing healthcare in Cameroon requires more than symbolic recognition or policy adjustments—it demands a reimagining of what constitutes legitimate medical knowledge, who is recognized as a healer, and how women’s voices and experiences inform healthcare design. When meaningfully integrated, traditional medicine offers not only cultural legitimacy but also a path toward more inclusive, affordable, and sustainable care [15, 16].

The Ministry of Public Health in Cameroon has acknowledged the vital role of traditional medicine, recognizing it as the primary healthcare provider for up to 80% of the population, a culturally trusted system for managing common and chronic ailments, and a key partner in achieving national health goals, particularly in rural and peri-urban communities [17–19]. Nonetheless, biomedical institutions continue to marginalize traditional practices, labeling them “unscientific” or “informal” [20], further entrenching institutional blind spots and undermining community trust.

This marginalization is compounded by gender dynamics. While women’s knowledge is preserved through oral traditions, apprenticeships, and experience, often closely tied to their roles in reproductive, maternal, and child health. As cultural custodians of knowledge regarding medicinal plants and holistic healing practices, women’s contributions are indispensable [21, 22]. However, patriarchal norms frequently devalue women’s roles relative to male healers, reflecting broader patterns of epistemic injustice [23, 24]. This situation creates a double marginalization for women traditional healers. While all traditional practitioners face systemic external barriers—including exclusion from formal healthcare institutions, lack of recognition by medical councils, and regulatory obstacles—these challenges are often compounded for women. The internal patriarchal norms can lead to greater stigmatization and more restricted access to resources and recognition even within their own healing communities, directly reflecting the kind of internal challenges that require a balanced perspective [25–27]. While the literature extensively documents the historical marginalization of traditional medicine, its continued use, and women’s critical roles as both practitioners and knowledge holders, a striking gap remains in empirical research on practical and ethical models for systematic integration—particularly those led by or centered on women. Existing studies affirm the cultural relevance and accessibility of traditional medicine and advocate for decolonized health approaches but rarely assess the outcomes of integration initiatives, explore gender-sensitive training programs for healers, or analyze the socio-economic benefits of recognizing women as traditional practitioners. Decolonisation is a lived, bodily experience rather than solely an intellectual process, and it also questions the notion of knowledge that is detached from the body [28, 29]. Policy frameworks tend to focus on recognition rather than on transforming power relations or ensuring equitable resource distribution.

This study aims to address these gaps by examining the role of traditional medicine in the lives of Cameroonian women, as both users and healers, and exploring how its integration into national health strategies can contribute to a more equitable and decolonized healthcare system. Drawing on feminist political economy and decolonial theory, this study employs a qualitative approach, using in-depth interviews, to investigate the socio-economic, cultural, and institutional dynamics that influence women’s healthcare choices and the positioning of traditional medicine in national discourse [30]. It aims to illuminate how strengthening indigenous health systems can enhance women’s health, autonomy, and agency while confronting the structural inequalities embedded in postcolonial health governance.

## Literature review

### Decolonizing healthcare: epistemic justice and Indigenous knowledge

The call to decolonize healthcare is fundamentally a call for epistemic justice. This reflects what theorists describe as epistemic injustice—the systematic devaluation and dismissal of certain forms of knowledge [20]. The historical marginalization of indigenous health practices is a form of epistemic violence that systematically devalues non-Western knowledge systems [31, 32]. In response, decolonial scholarship not only underscores the significance of indigenous knowledge systems in challenging the dominance of Eurocentric epistemologies [33, 34], but also offers a transformative potential. This critical perspective envisions a future built on a “pluriversal” approach, where diverse medical systems can coexist without hierarchy [35, 36], moving beyond token inclusion toward genuine systemic transformation.

### Conceptualizing health and value in traditional medicine

Central to the decolonization of healthcare is the recognition that health is conceptualized differently outside the biomedical model. Many indigenous paradigms are built on holistic foundations, aligning with African epistemologies that view health as a state of equilibrium between the individual, community, ancestors, and environment [37]. This understanding is crucial as it broadens the concept of the value of traditional medicine. It is not merely clinical but a multidimensional construct, encompassing therapeutic efficacy, cultural resonance, social empowerment, spiritual well-being, and economic accessibility. Recognizing this broad, lived concept of value is essential for assessing the proper role of traditional medicine in people’s lives.

### Theoretical framework: feminist political economy of health (FPEH)

To analyze the intersecting dynamics of gender, power, and health, this study employs a Feminist Political Economy of Health (FPEH) framework [38, 39]. This lens reveals how gender, power, and economic structures intersect to shape women’s health experiences, particularly where traditional knowledge is marginalized. The FPEH framework posits that the subordination of women is not only economic but also epistemological, rendering it uniquely suited to investigating the double marginalization faced by women healers [20, 27, 40]. This study used FPEH to examine how women’s roles as both users and practitioners of traditional medicine are shaped by, and in turn shape, the broader political and economic landscape of healthcare in Cameroon, highlighting the significance and relevance of this framework in our research.

### The formalization of traditional medicine: law No. 2024/018

The legal status of traditional medicine in Cameroon was officially formalized with the enactment of Law No. 2024/018 on December 23, 2024 [41]. This pivotal legislation moves the sector from the margins to a recognized component of the national healthcare apparatus. Its foundation rests on three key pillars: providing a formal definition, creating a regulatory framework, and mandating collaboration with modern medicine.

A cornerstone of the law is its definition of traditional medicine as a comprehensive body of knowledge, skills, and practices grounded in indigenous experience. This formally recognizes the legitimacy of methods incorporating plants, natural substances, and spiritual elements for maintaining health and treating illness. This formal recognition validates the long-standing practices of traditional medicine practitioners and acknowledges their significant contributions to healthcare. The new regulatory framework it establishes paves the way for future standards in training and ethical practice. Most significantly, the law mandates collaboration between traditional and modern medicine. By creating a legal foundation for integration rather than opposition, it positions them as complementary approaches to achieving public health goals in Cameroon.

### Central research aim

To analyze the experienced value of traditional medicine in the lives of Cameroonian women (as both users and healers), and to explore how these insights can inform the development of inclusive, culturally responsive, and decolonized health systems.

### Research questions

#### Main Question

- How can traditional medicine, as practiced and valued by women in Cameroon, contribute to the development of inclusive and decolonized health systems?

#### Sub-questions

- How do Cameroonian women experience, practice, and value traditional medicine within their cultural, social, and economic contexts?
- What institutional, policy, and systemic barriers hinder the integration of women-led traditional medicine into national healthcare systems, and how can these be addressed to promote inclusive, gender-responsive healthcare reform?

## Methods

### Research design

This manuscript reports on the findings from the initial qualitative phase of a larger mixed-methods study. The current study employs a qualitative, exploratory design, uniquely grounded in decolonial and feminist epistemologies [42, 43]. This distinctive qualitative approach aimed to conduct an in-depth investigation into how traditional medicine, as practiced and experienced by women in Cameroon, can inform more inclusive and decolonized health systems.

By centering the lived experiences and indigenous knowledge of women as both practitioners and users, the study challenges biomedical hegemony. The decolonial lens acknowledges the plurality of health systems, while a feminist approach highlights women’s agency and the intersection of gender, power, and access to healthcare. This framework enabled a relational and ethically reflexive inquiry, valuing storytelling and dialogue to co-create pathways for equitable health systems. Importantly, to ensure comprehensive and transparent reporting of our qualitative process and findings, this study adhered to the Consolidated Criteria for Reporting Qualitative Research (COREQ) guidelines [44].

This manuscript focuses specifically on the findings from traditional healers and users to provide an in-depth analysis of their lived experiences and the cultural significance of traditional medicine. The data gathered from biomedical and policy stakeholders, which addresses institutional perspectives, will be analyzed separately and presented in a subsequent publication.

### Study setting and context

The geographical scope of this study was strategically designed to encompass three diverse regions of Cameroon: the Centre, North West, and South West. This selection aimed to provide a comprehensive understanding by including contexts that varied in cultural practices, geographical features, healthcare accessibility, and the specific roles of traditional healing. The Centre Region offered insights into mixed urban-rural healthcare environments, while the North West, a conflict-affected highland, highlighted reliance on traditional medicine in underserved areas. The South West, a biodiverse coastal region, showcased strong ancestral and plant-based healing legacies.

### Participants

Participants were recruited using a combination of purposive and snowball sampling to ensure the deliberate inclusion of diverse, contextually relevant experiences. The specific application of these methods varied across the three participant groups:

- Users of Traditional Medicine: Women with personal experience using traditional medicine were primarily identified through purposive sampling at community centers, women’s associations, and local markets in the study regions. This strategy was designed to capture a range of ages, backgrounds, and health experiences. Following initial interviews, snowball sampling was employed, as participants were asked to refer other women in their community who also met the inclusion criteria.
- Traditional Healers: A similar dual approach was used to recruit practitioners. Purposive sampling initially targeted known healers with different specializations (herbalists, spiritual healers, midwives). Subsequently, snowball sampling was essential for accessing unregistered or lesser-known healers, especially those operating in rural and conflict-affected regions where formal records are limited and access is facilitated through established community trust networks.
- Biomedical and Policy Stakeholders: This group was recruited exclusively through purposive sampling. Individuals were strategically identified and contacted based on their professional roles (e.g., healthcare professionals, hospital administrators, ministry officials) to ensure that key institutional and policy perspectives on the integration of traditional medicine were included in the study.

It’s important to note that while participants were recruited into three distinct categories (‘users,’ ‘healers,’ ‘stakeholders’) for analytical clarity, these identities are not mutually exclusive and are often fluid. For the purpose of this study, ‘stakeholders’ were defined as individuals holding positions within formal institutions (e.g., government, biomedical facilities). Healers who were also community advocates or organizers were not categorized as separate entities, but rather as practitioners, with their advocacy work analyzed as a key dimension of their practice. The intersectionality of these roles is itself considered a significant area of inquiry within our findings.

The study sample consisted of 30 participants, strategically selected to represent a range of perspectives across various healthcare systems and experiences. This sample size was considered appropriate given the depth of inquiry and the study’s goal of surfacing diverse and underrepresented voices.

- 15 Cameroonian women (aged 18–65) with personal experience using traditional medicine, drawn from the three study regions.
- 10 traditional medicine practitioners, including herbalists, spiritual healers, and community-based midwives, were interviewed for their insights into indigenous health practices related to women’s health. The roles of these informal practitioners frequently intersect, which is crucial to their operation at the community level. This intersectionality means that a single practitioner often embodies multiple functions. For example, a traditional midwife is typically also an herbalist who provides specific plants for prenatal and postpartum care; a nutritional counselor who advises on diet; and a spiritual guide who performs rituals to protect the mother and child. This multifaceted role ensures that a woman receives holistic, continuous, and trusting care from one individual deeply embedded in her community, which contrasts with the fragmented system of separate specialists. Acknowledging this integration of roles is key to understanding the value of community-based traditional healthcare.
- 5 biomedical and policy stakeholders, including healthcare professionals, hospital administrators, and officials involved in health planning and regulation. Their insights provided valuable perspectives on the intersection of traditional and modern healthcare systems, as well as institutional views on the integration and recognition of traditional medicine within national health frameworks.

### Participant inclusion and exclusion criteria

Participants were selected through purposive and snowball sampling. This selection process was guided by the study’s focus on inclusivity and diversity, aiming to capture a comprehensive range of perspectives and experiences.

### Inclusion criteria

Eligible participants met the following requirements:

- Cameroonian women aged 18–65 with direct, lived experience of utilizing traditional medicine.
- Traditional healers actively engaged in any form of traditional healing practice for a minimum of one year.
- Stakeholders (e.g., policymakers, biomedical health professionals, NGO representatives) with a professional background or active involvement in women’s health or traditional medicine, whether in healthcare delivery, policy development, or advocacy.
- Ability and willingness to provide informed consent (either written or verbal) and participate in interviews or discussions.

### Exclusion Criteria

Participants were excluded from the study if they met any of the following conditions:

- Lack of direct experience with traditional medicine, defined as either personal use or professional practice.
- Individuals under 18 years of age, primarily to simplify ethical considerations by avoiding the complexities of guardian consent for community-based engagement.
- The presence of language barriers, severe illness, or emotional distress would impede their effective participation during fieldwork.
- As an ethical precaution, individuals holding specific government positions responsible for regulating traditional medicine were not recruited. This decision was made by the research team to avoid placing potential participants in a conflict of interest where speaking openly could lead to perceived or actual professional repercussions.

### Data collection

To capture the rich, context-specific, and gendered dimensions of traditional medicine in Cameroon, this study employed two qualitative data collection methods. This comprehensive approach, designed to align with the study’s decolonial and feminist framework, aimed to ensure the inclusion of voices and experiences often marginalized in mainstream health research. It was a testament to the thoroughness and depth of the research process, which valued diverse perspectives and centered on each participant’s unique contribution.

Individual semi-structured interviews were conducted with Cameroonian women (both users and practitioners of traditional medicine), traditional healers, biomedical professionals, and policy stakeholders. This method facilitated a deep exploration of personal experiences, cultural understandings, health-seeking behaviors, and the social and emotional meanings associated with traditional healing. The semi-structured format ensured thoroughness by consistently addressing key thematic areas while allowing for the emergence of new insights.

Data collection transpired over four months, from January to April 2025, encompassing both urban and rural communities across the three selected regions in Cameroon. Interviews conducted by coauthors took place in participants’ homes, healing spaces, or local community centers based on their preferences and comfort. Conversations were conducted in English, French, or relevant local dialects, with the assistance of trained translators when necessary. This inclusive approach aimed to foster a culturally respectful environment where all participants felt equally valued.

Participants were recruited using purposive and snowball sampling techniques, with a particular focus on reaching women and traditional healers in remote or conflict-affected areas who might not be formally registered. Interviews continued until thematic saturation was achieved, indicating that no new significant information or themes were emerging from the data.

### Participant observation for contextual understanding

In addition to interviews, limited participant observation was conducted to build rapport and gain a deeper contextual understanding that would inform the interview process. Where culturally appropriate and ethically permissible, this included observing interactions at community markets and informal community gatherings, as well as the general atmosphere of healing spaces.

The primary purpose of these observations was to contextualize the narratives shared in the interviews, rather than for a separate thematic analysis. Field notes were kept with careful attention to the researchers’ positionality and ethical boundaries, focusing on insights that would aid in interpreting the interview data. Throughout this process, researchers ensured that observation did not violate sacred or private spaces, and consent was sought from community leaders where applicable.

Therefore, the thematic findings presented in this manuscript are drawn exclusively from the analysis of the interview data.

### Ethical considerations

The study received formal ethical approval from the Eglise Evangelique Lutherienne du Cameroun (EELC) and Hospital Protestant de Ngaoundere (Ref. No: 030/EELC/OSEELC/HPNDERE/DIR/24). All research phases were guided by the core principles of ethical sensitivity, reciprocity, transparency, and cultural humility, prioritizing the dignity, safety, and agency of all participants.

### Informed consent process

Informed consent was a mandatory prerequisite for all participants. Prior to the consent discussion, each potential participant was provided with a detailed Information Sheet explaining the study’s purpose, procedures, confidentiality measures, potential risks and benefits, and their explicit right to withdraw at any time. For participants with limited literacy, the researcher read this information sheet aloud to ensure complete comprehension before proceeding.

To ensure a culturally responsive manner, a flexible process utilizing both written and verbal consent was adopted. Written consent was the standard for stakeholders in formal institutions and other literate participants. Verbal consent was offered as an essential alternative for those in community settings or with limited literacy.

During the consent process, permission to audio-record the interview was requested separately from the general consent to participate in the study. Participants were informed that they could decline to be recorded and still participate in the study (in which case, detailed handwritten notes would be taken). For this study, all participants who were interviewed gave their express permission for audio recording to be used.

When verbal consent was obtained, the researcher documented the agreement on a dedicated form, signed by both the researcher and, when possible, a witness present during the discussion.

### Confidentiality and data management

To ensure participant confidentiality, all identifying details were removed from transcripts and research materials, and pseudonyms, often chosen in consultation with participants, were used in all written outputs. All interview recordings and transcribed data were stored securely on encrypted devices, with access restricted to the research team only.

### Community engagement and vulnerable populations

To ensure respectful and contextually appropriate engagement, the research team collaborated with local mediators, elders, and cultural brokers. These intermediaries were vital for navigating cultural norms, gender dynamics, and language barriers. Particular care was exercised when engaging with vulnerable populations, specifically women residing in conflict-affected regions and traditional healers whose practices could involve sensitive information, to ensure their participation was both safe and meaningful.

### Data analysis

This study employed thematic analysis, as outlined by Braun and Clarke’s (2006) six-phase framework, to identify, analyze, and report patterns of meaning across the data set [45]. This approach was selected due to its flexibility and alignment with feminist and decolonial epistemologies, which prioritize participant voices, especially those of marginalized communities such as Cameroonian women and stakeholders involved in or reliant on traditional medicine.

1. Familiarization with the Data: All interviews were transcribed verbatim. The transcripts were read repeatedly to ensure deep immersion in the content. Initial ideas were noted in the margins and in a research journal to guide early thoughts on potential codes and themes. This stage allowed the researcher to engage holistically with participants’ narratives and contextual subtleties.
2. Generating Initial Codes: The data were coded inductively, identifying meaningful segments of text that related to the research questions and also facilitated theme visualization. Coding was done manually and through NVivo software. Codes captured the essence of participant experiences and reflections concerning traditional medicine, its uses, challenges, and cultural significance. Codes reflected recurring ideas, practices, metaphors, and resistances articulated by participants.
  *Example Codes*: “Connected to roots,” “Hospitals too expensive,” “Healing through prayers,” “Respected without formal education,” “Traditional medicine is ours.”

3. Searching for Themes: Codes were organized into potential themes and subthemes. Patterns were identified across interviews and focus groups, allowing for the clustering of codes with shared meanings. This phase involved grouping codes to reflect broader concepts such as identity, accessibility, and empowerment.
  *To illustrate the analytical process*,* consider the following examples from our coding to theme development*: The code “Connected to roots” directly informed the subtheme “Reclaiming Ancestral Knowledge,” which contributed to the overarching theme of “Traditional Medicine as a Source of Cultural Identity and Empowerment.” Similarly, the code “Respected despite no training” highlighted “Women’s Authority in Health Practices,” further reinforcing the same theme.
  In another instance, the code “Hospitals too expensive” pointed to the subtheme of “Affordability and Availability,” which was central to the theme “Traditional Medicine as an Accessible and Responsive Health Resource.” Finally, the code “Healing of soul and body” provided direct support to the subtheme “Holistic and Culturally Attuned Care,” also contributing to the theme of traditional medicine as a responsive and accessible health resource.

4. Reviewing Themes: The themes were reviewed across the entire data set to ensure internal coherence and distinctiveness. Extracts within each theme were compared to validate their fit. Some themes were refined or merged to strengthen conceptual clarity.
  *Example: *Codes initially categorized under “healing and spirituality” and “ancestral knowledge” were refined and merged into the subtheme “Reclaiming Ancestral Knowledge and Practices.”

5. Defining and Naming Themes: Themes and subthemes were clearly defined, with attention to their contribution to the overall research questions. The names were selected to reflect the socio-cultural and political meanings embedded in the participants’ narratives. For instance, “Traditional Medicine as a Source of Cultural Identity and Empowerment” encapsulates the role of traditional healing as a medium of cultural continuity, feminine authority, and resistance against biomedical dominance.
6. Producing the Report: The themes were developed into a coherent analytic narrative, addressing the study’s central research aim. Each theme was illustrated with vivid, context-rich excerpts from participant interviews and group discussions. The findings were situated within broader scholarly debates on decolonizing healthcare and inclusive health systems. This systematic, reflective, and iterative analysis ensured that the findings were rooted in participants’ realities and offered valuable insights into how traditional medicine, as practiced by and for Cameroonian women, can inform inclusive and decolonized health reforms.

## Findings

The findings presented in this section are drawn from our thematic analysis of interviews with traditional healers and users. A central ‘golden thread’ weaving through their narratives is the profound role of traditional medicine in shaping and reflecting on women’s self-identity, as healers, cultural custodians, and Cameroonians navigating postcolonial health systems. This overarching insight is explored through four primary themes: (1) Traditional Medicine as a Source of Cultural Identity and Empowerment, (2) Traditional Medicine as an Accessible and Responsive Health Resource, (3) Marginalization within the Formal Health System, and (4) Pathways Toward Inclusive and Decolonized Health Systems. Together, these themes illuminate the lived realities, cultural meanings, and systemic challenges faced by Cameroonian women about traditional medicine.

### Traditional medicine as a source of cultural identity and empowerment

This theme highlights how traditional medicine is perceived not only as a healing practice but also as a vital expression of cultural identity and women’s agency. For many participants, engaging with traditional healing was a way of reconnecting with ancestral legacies and reaffirming their place within the socio-cultural fabric of their communities.

#### Reclaiming ancestral knowledge and practices and forging Self-Identity

This subtheme focuses on how traditional medicine serves as a conduit for cultural continuity and pride. Participants shared personal narratives of intergenerational learning and spiritual connection through traditional healing knowledge. For example, Participant 3, a traditional healer from the North West Region, stated, " *Traditional practices are not just about healing; they represent who we are. They give us a sense of identity*,* of being safe*,* and of having control over our own choices…When I use the herbs … my grandmother taught me*,* I feel connected to my roots. It is more than healing; it is identity.”* Similarly, Participant 12 from the South West Region remarked, *“Western medicine always felt foreign to me — like a language I couldn’t fully speak*,* with treatments that sometimes overlooked the spirit*,* the heart*,* and the deep connections we have with the land. But with traditional medicine*,* everything feels familiar. I […] understand the language of the plants*,* the prayers*,* the rhythms of healing passed down from our mothers and grandmothers. It is ours — woven into our stories*,* our daily lives*,* and our very being. As women healers*,* we […] carry this sacred knowledge*,* nurturing not just bodies but souls*,* reclaiming the power that colonial histories tried to take away.”* These perspectives underscore the importance of cultural relevance and familiarity in health-seeking behaviors, especially in regions where traditional systems are deeply embedded.

#### Women’s authority in health practices

This subtheme captures how women assume and retain positions of authority within traditional healthcare spaces, often commanding respect and recognition from their communities despite systemic exclusion from formal biomedical structures. Participant 7, a midwife and herbalist from the Centre Region, noted, *“In our community*,* women have always been the keepers of healing — the guardians of ancient knowledge passed down through generations. We know the plants*,* the rituals*,* the stories that restore health and balance. Even when men rely on their own methods or modern medicine*,* they come to us when those ways fail. They […] seek our wisdom*,* our care*,* and our hands. Our authority in healing is quiet but undeniable — it is a power rooted in trust*,* experience*,* and the deep bonds we hold with our people.”* Participant 10, a traditional practitioner from the South West Region, added, " *For us*,* traditional healing means more than just medicine — it’s about our roots*,* our safety*,* and the power to decide for ourselves. Even though I […] didn’t go to school for medicine*,* women like me are respected because we heal with what we know.*” These insights reveal a feminized space of power and respect within the informal health sector, where experiential and indigenous knowledge is highly valued, challenging biomedical epistemic hierarchies.

### Traditional medicine as an accessible and responsive health resource

This theme emphasizes the practical value of traditional medicine as a health resource that is both accessible and attuned to the everyday realities of Cameroonian women. In contexts where factors such as financial constraints and geographical distance, among others, make biomedical services inaccessible, traditional medicine emerges as a trusted and responsive alternative that meets not only physical health needs but also emotional, spiritual, and communal concerns.

#### Affordability and availability

This subtheme highlights how traditional medicine remains financially accessible and embedded in the local community, particularly during periods of crisis or systemic collapse. Participant 6, a user from the Centre Region, explained, *“Hospitals are too expensive for many of us—too far*,* too crowded*,* and sometimes too cold and impersonal. But I can go to Mama Healer’s place anytime. She knows me*,* my family*,* and my struggles. She listens patiently*,* and she […] gives me what I need without worrying about the cost. Her care is always within reach*,* filled with warmth and understanding. For many in our community*,* she is not just a healer*,* but a lifeline — affordable*,* available*,* and deeply trusted.”* Participant 11, a user from the North West Region, reflected on how conflict forced communities to depend on traditional remedies: *“Especially during the crisis*,* when […] pharmacies were closed and hospitals overwhelmed*,* we relied only on the herbs and remedies nature provided. The plants never shut their doors — they […] were always there*,* growing freely around us*,* ready to heal. Our communities turned to us because we were available*,* affordable*,* and connected to the land in a way that no clinic or pharmacy could be. In those hardest times*,* traditional medicine wasn’t just a choice — it was a lifeline*,* a source of hope and survival that reminded us of our strength and resilience as women healers.”* These statements emphasize traditional medicine as an economically viable and culturally embedded alternative that provides continuity of care when formal services are unavailable.

#### Holistic and culturally attuned care

This subtheme reflects participants’ appreciation for the comprehensive and culturally meaningful ways in which traditional healers engage with illness and well-being, often addressing issues that biomedicine overlooks. Participant 15, a user from the South West Region, shared, “*When I went to the hospital*,* they treated only the fever… hmm. But the healer […] prayed*,* asked about my dreams*,* and treated my soul too.*” Participant 4, a healer from the Centre Region, noted, “*Traditional healing is about the whole person*,* not just disease. […] It’s not just treating illness — traditional healing takes care of your mind*,* your spirit*,* your connection to others. That’s what makes women feel seen and safe…That’s why women trust it*.” These perspectives underscore how traditional healing addresses spiritual, emotional, and psychosocial dimensions of health, earning trust due to its empathetic and culturally resonant nature.

### Marginalization within the formal health system

While participants acknowledged the value and resilience of traditional medicine, many also reflected on the structural and institutional barriers that continue to undermine its legitimacy. This theme captures how traditional medicine—particularly as practiced by women—has been marginalized within formal health systems and national policy frameworks.

#### The persistent struggle for institutional recognition

This subtheme underscores how traditional healers have historically operated operate in legal and professional grey zones, with little protection or institutional support. These experiences, reflecting decades of systemic neglect, highlight the profound need for the very legal changes that are only now beginning to take shape. Participant 9, an herbalist from the South West Region, stated, *“They call us witches*,* or say what we do is unscientific — dismiss us with a shrug or a smirk. But when the clinics fail*,* when the medicines don’t work*,* or when someone’s spirit is breaking from grief or mental illness*,* they come to us. They seek our hands*,* our words*,* our knowledge. They don’t see us in their systems*,* but they find us when they’re lost.*” Participant 13, a traditional healer from the North West Region, added, “*“There […] are no spaces for us in health programs. Policies systematically ignore*,* marginalize*,* and devalue the knowledge and practices we’ve cultivated for generations as healers. It’s as if our contributions to community health don’t exist in their eyes.*” Furthermore, Participant 19, a community organizer and herbalist from the North West Region, stressed, “*We need laws that protect us and training that include us. Health policies should start […] with the people*,* not just the ministries.*” These remarks highlight systemic marginalization, epistemic injustice, and the ongoing struggle for formal acknowledgement of their invaluable contributions. It is precisely this long-standing struggle, articulated so powerfully by the participants, that the recent Presidential Decree n° 2024/018 of December 23, 2024, aims to address by formally recognizing the practice of traditional medicine in Cameroon.

#### Gendered exclusion in health policy and dialogue

This subtheme reveals the gendered exclusion and systemic silencing of women healers within health governance and decision-making spaces. Participant 2, a midwife from the Centre Region, observed, *“Male officials sit in rooms*,* writing policies and making decisions about health — but they never ask the women who actually do the healing work. We are left out of the conversations*,* ignored in the meetings*,* invisible in the reports. They speak as if they know our communities better than we do*,* as if healing is something that happens only in hospitals*,* not in homes*,* markets*,* and hearts where we work every day. It’s not just exclusion — it’s erasure.”* Participant 14, a healer from the North West Region, recounted her experience: *“I went to a health meeting once*,* hoping to share what I’ve learned from years of healing in my community. But the moment I said I was a healer*,* they dismissed me — their faces changed*,* their eyes glazed over*,* as if my voice no longer mattered. They didn’t want to hear about herbs*,* rituals*,* or the way we care for people’s souls*,* not just their symptoms. I felt small*,* like I didn’t belong in a space that should have welcomed every kind of knowledge — especially the kind that’s kept our people alive for generations.”* These testimonies illustrate the disconnect between male-dominated formal leadership and grassroots expertise, exposing how women’s embodied, experiential healing knowledge is often disregarded as invalid or unauthoritative in official settings.

### Pathways toward inclusive and decolonized health systems

Despite the challenges, participants also articulated powerful visions for more inclusive, respectful, and collaborative healthcare futures. This theme highlights the potential of traditional medicine to serve as a foundation for decolonizing healthcare in Cameroon, especially when women’s knowledge and leadership are centered.

#### Recognition and regulation of traditional medicine

This subtheme emphasizes the urgent need for formal recognition, legal protection, and institutional integration of traditional healing within national health systems. Participants expressed a desire to work alongside biomedical systems, not replace them, in ways that affirm their knowledge and enhance patient care. Participant 8, a healer from the Centre Region, stated, *“If the government could certify us*,* recognize our knowledge*,* and give us space in health centres*,* it would change everything. It would not only validate the work that so many women have done quietly for generations*,* but it would also help countless women who currently have nowhere else to turn. Certification would bring trust and safety*,* and opening spaces for us would mean healing becomes truly inclusive — embracing both modern medicine and the wisdom of our traditions. This recognition could empower women healers and transform the health of entire communities.”* Participant 1, an herbalist from the South West Region, added, “*There should be a law that protects our knowledge and lets us work alongside hospitals.*” Participant 23, a healer from the Centre Region, highlighted, *“We are never invited to health meetings. Our names aren’t on the lists*,* our voices aren’t in the rooms. Yet*,* when people fall sick — truly sick*,* when hospitals turn them away or can’t explain what’s wrong — they come to us. They trust our hands*,* our knowledge*,* our way of healing. We carry generations of wisdom*,* but still*,* we are treated like we don’t exist. All we ask is to be seen*,* heard*,* and respected — not just by our communities*,* but by the systems that claim to care for them.”* These voices express a hope for systemic transformation that grants dignity, recognition, and improved access for patients, while empowering healers, especially women.

#### Collaborative models of care

This subtheme points to the importance of dialogue and partnership between traditional and biomedical sectors, particularly through community-based initiatives and referral systems. Participant 5, a policy officer from Yaoundé, articulated a transformative vision: “Imagine a clinic where doctors and traditional healers work together. That would be true healing.” Participant 16, an NGO representative from the Centre Region, called for bidirectional learning: *“We need training for both sides — so nurses and doctors can learn about our medicine*,* our herbs*,* and the ways we care for the whole person*,* not just the illness. And so we can understand their science*,* their treatments*,* and the ways hospitals work. Only then can we truly collaborate*,* bridging the gap between tradition and modern healthcare. This kind of respect and shared knowledge could break down the walls of distrust*,* […] decolonize the healthcare system*,* and create a space where all forms of healing are valued and work together for the wellbeing of our communities.”* Participant 27, a healer and birth attendant from the South Region, underscored, “*Doctors and healers men and women must sit at the same table—not to fight*,* […] but to learn. If we work together*,* the people will benefit*.” These perspectives support a model of intercultural health education and practice where dialogue and mutual respect replace dominance and marginalization.

## Discussions

This study explores the vital role of traditional medicine in the lives of Cameroonian women, revealing its significance beyond mere healing practices. Our findings highlight how traditional medicine serves as a fundamental aspect of cultural identity and empowerment, offers a holistic and culturally attuned approach to care, faces persistent barriers to legitimacy and institutional recognition, and presents clear pathways toward inclusive and decolonized health systems. These themes are critically examined by applying the Feminist Political Economy of Health (FPEH) lens established earlier [39], which illuminates the intersecting impacts of gender, power, and economic structures on women’s health and healing within a context where traditional knowledge is often marginalized.

### Traditional medicine as a source of cultural identity and empowerment

For many Cameroonian women, traditional medicine is a core component of their cultural identity, ancestral continuity, and gendered authority. Our narratives reveal that traditional medicine operates within a framework of embodied knowledge, spirituality, and social recognition that empowers women both as healers and cultural custodians.

Reclaiming ancestral knowledge demonstrates how traditional medicine functions as a living archive of indigenous wisdom, passed down across generations of women. Participants emphasized the emotional and spiritual resonance of practicing what their mothers and grandmothers had taught them, describing healing as a way of reconnecting with their roots. This act of reclaiming knowledge is a powerful form of cultural resistance that challenges the dominance of Eurocentric epistemologies, as outlined in our literature review [33, 34]. In a context where colonial legacies have long privileged Western biomedical models, the revival and valuing of traditional medicine represent a powerful form of cultural resistance and epistemic self-determination [46]. One participant emphasized that the practice was ultimately about identity, not simply healing. This sense of rootedness directly confronts the historical marginalization of indigenous practices. This marginalization is a clear example of the epistemic violence previously described [31, 32].

Traditional medicine also emerged as a space where women exercise authority, skill, and leadership. Several participants positioned themselves as primary health providers within their communities, challenging dominant narratives that often portray traditional healers as male or indigenous knowledge as inferior. The transmission of this knowledge, often through female lineages [2], underscores the central role of women in preserving these cultural practices. The role of women as knowledge holders and community healers complicates Western biomedical paradigms that often exclude non-credentialed practitioners and supports the feminist and decolonial critiques of health systems outlined earlier [47, 48]. The testimony of one participant revealed a community-based system where legitimacy and respect are derived from practical healing outcomes and inherited knowledge, rather than from formal medical education. This highlights the importance of recognizing not just traditional medicine itself, but the gendered structures within which it is practiced. Supporting women healers and their knowledge systems is essential for both cultural preservation and creating equitable, decolonized health infrastructures.

Beyond its cultural significance, traditional medicine was described by participants as a vital and pragmatic alternative to formal healthcare systems [3, 17]. Its accessibility—both geographically and financially—combined with its holistic, culturally attuned approach to healing, positions traditional medicine as a key component in addressing healthcare inequalities in Cameroon. This theme underscores how traditional medicine not only fills gaps left by the formal health sector but also offers a more personalized and community-rooted model of care. Many participants emphasized the economic and logistical barriers to biomedical care, particularly in rural or conflict-affected areas, turning instead to traditional remedies embedded in their daily lives. One participant underscored the resilience of local knowledge systems by describing how, in the absence of formal pharmacies, the natural environment served as a constant and reliable source of medicine. This encapsulates the enduring relevance of local knowledge systems in ensuring healthcare continuity when formal systems fail. It also challenges assumptions that equate “modern” with “effective,” highlighting the resilience and resourcefulness of community-based care systems.

While emphasizing the empowerment women derive from their roles in traditional medicine, a balanced perspective necessitates acknowledging potential internal challenges that go beyond gender. For instance, access to specialized healing knowledge or control over rare medicinal resources might be vested in specific lineages or elder practitioners, creating internal hierarchies based on seniority or kinship rather than gender. Furthermore, while women often dominate practices related to reproductive health, men may hold more authority in other domains, such as specialized herbalism or spiritual healing, reflecting the complex and diverse social fabric of these systems.

Our findings reveal the limitations of rigid analytical categories and highlight the profound intersectionality of our participants’ roles. The neat distinctions between ‘healer,’ ‘user,’ and ‘stakeholder’ often dissolved in the lived realities of the women we spoke with. These realities, often characterized by socio-economic challenges, cultural norms, and gender roles, influenced the roles of the participants. Several traditional healers, for instance, were also powerful community organizers and advocates, effectively blurring the lines between a practitioner and a community stakeholder. This fluidity is not a methodological challenge but a key finding in itself. It demonstrates that leadership in traditional medicine is a holistic practice that often extends beyond healing to encompass community-building, social advocacy, and political resistance, reinforcing the need for health systems to engage with practitioners not just as clinicians, but as integral community leaders.

### Holistic and culturally attuned care

Participants also highlighted the spiritual, emotional, and social dimensions of traditional healing, contrasting them with the perceived impersonal and fragmented nature of biomedical care. Traditional healers were seen not just as providers of remedies, but as caretakers of the whole person—addressing psychological distress, spiritual imbalance, and social disharmony alongside physical symptoms. This finding provides a clear illustration of the holistic African epistemologies discussed previously, which view health as an integrated state of equilibrium [37]. Highlighting a key difference from a conventional clinical encounter, a participant shared that their healer’s treatment included spiritual and psychological dimensions, such as prayer and dream analysis, to care for their “soul.” Such testimonies illustrate how traditional medicine offers more than symptomatic relief; it provides a culturally meaningful framework for understanding and navigating illness and recovery. These findings support calls for more culturally responsive health systems that validate local worldviews and patient narratives. Incorporating such holistic models could foster trust, improve uptake, and reduce disparities—particularly for women who often experience intersecting forms of marginalization in healthcare.

### Barriers to legitimacy and institutional recognition

Despite its deep embedment in the everyday lives of many Cameroonian women, the study highlights persistent challenges in gaining formal recognition and integration within the national health system. This underscores the tension between locally rooted practices and official state health policies, revealing how colonial legacies and current political structures continue to marginalize traditional knowledge—particularly when embodied and transmitted by women.

Several participants expressed frustration at the lack of respect and acknowledgment for their knowledge from authorities, healthcare professionals, and academic institutions. Traditional healers often perceive themselves as “primitive” or “unscientific,” despite their long-standing roles and practical expertise in community health. This experience is a direct manifestation of the epistemic injustice defined in our conceptual framework [20]. As one female practitioner articulated, this creates a paradox of being essential to community health while remaining invisible within official health policy discourse. This is not merely individual exclusion but a broader structural issue impeding the development of inclusive and equitable health systems.

Another significant barrier relates to gendered power dynamics in health governance and policy-making. Participants emphasized how women—despite being central to the practice and transmission of traditional healing—are often excluded from decision-making processes at institutional levels. This form of gendered exclusion is embedded in wider patriarchal structures where both women’s experiences and their knowledge are devalued [25]. According to a health worker and activist, the marginalization of traditional knowledge is fundamentally a gendered issue. She argued that it is not the knowledge itself that is rejected, but rather the authority of its primary holders—women—that is systematically ignored. This illustrates the need to recognize not only the content of traditional knowledge but also the power relations surrounding it. Decolonizing healthcare in Cameroon requires a feminist lens that values and amplifies the leadership and voices of women who are custodians of healing traditions. These findings highlight the urgent need for structural reforms in how knowledge is validated, recognized, and integrated. A truly decolonized and inclusive healthcare system cannot be built without addressing both epistemic injustice and gender-based power inequalities.

### Pathways toward inclusive and decolonized health systems

This final theme captures participants’ visions, strategies, and recommendations for transforming Cameroon’s health landscape into one that is inclusive, gender-sensitive, and culturally rooted. Far from presenting themselves as victims of marginalization, many women voiced powerful ideas for how traditional medicine could be legitimized and integrated into formal healthcare systems in ways that respect both indigenous knowledge and the needs of diverse communities.

Many participants emphasized the potential for collaboration between biomedical and traditional systems, provided it is built on mutual respect rather than domination or tokenization. Women advocated for dialogue, knowledge-sharing platforms, and opportunities to learn from one another to improve patient outcomes and foster culturally attuned care. As one traditional midwife stressed, integration must not mean assimilation or erasure of traditional practices, but rather recognition of their unique contributions. Creating collaborative spaces where knowledge systems coexist on equal footing is a critical step in decolonizing healthcare, a vision that directly reflects the WHO Traditional Medicine Strategy and the ‘pluriversal’ approach outlined earlier [5, 35, 36].

In addition to professional collaboration, participants pointed to the importance of policy-level change. They argued that integration should not be imposed top-down but informed by grassroots realities and the lived experiences of women who use and practice traditional medicine. Many respondents were already involved in advocacy, community mobilization, or local associations working toward the recognition and regulation of traditional healing. These perspectives reflect a broader desire for participatory governance, where women’s voices are included in shaping the future of healthcare in Cameroon. A decolonial approach, as envisioned by the participants, involves transforming not only institutions but also power dynamics—placing women, culture, and community at the center of health system reform. This emphasis on systemic transformation rather than token inclusion is a key tenet of the decolonial frameworks previously discussed [35, 36] and emphasizes the need for systemic transformation rather than token inclusion [49, 50]. The agency and resistance embodied by women healers [51, 52] are central to this transformative process. This theme thus concludes by emphasizing agency, hope, and collective action, envisioning a future where traditional medicine is valued as a vital, living practice capable of healing both bodies and systems.

### Feminist political economy of health perspective

Applying the Feminist Political Economy of Health (FPEH) framework [38, 39], our findings show how gender, power, and economic structures intersect to shape women’s health experiences, particularly where traditional knowledge is marginalized. In Cameroon, traditional medicine is not just a healing tool but a practice deeply embedded in economic, political, and cultural dimensions that profoundly impact women’s agency and well-being.

Traditional medicine occupies a complex space as both an economic resource and a cultural practice. For many women, especially in rural and marginalized communities, it’s a crucial lifeline supporting their physical, emotional, and economic survival. The intersection of gender and economy is evident in how women engage with, practice, and rely on traditional healing methods daily. Women in rural areas often face limited access to formal healthcare due to geographical, financial, and socio-political barriers. The high cost of biomedical care and lack of infrastructure in remote areas drive women to traditional healers and herbal remedies [26, 27]. These practices are often more affordable, accessible, and culturally congruent, allowing women to seek care within their communities from trusted individuals—often other women healers.

From an FPEH perspective, this reliance reflects a broader socio-economic structure where women, particularly in rural and low-income contexts, are often excluded from formal economic systems that dictate healthcare provision. Traditional medicine thus serves as a vital economic resource, offering women self-care and income generation. Many women are involved in the production and sale of medicinal herbs, contributing to household economies and gaining recognition and authority within their communities.

However, the economic value of traditional medicine is frequently undermined in official economic and health policies. Women practitioners are rarely recognized as formal health workers or compensated for their critical services. This lack of institutional recognition is tied to broader gendered economic disparities that limit women’s access to formal employment and leadership roles in health systems [53, 54]. The cost-effectiveness and community-based nature of traditional medicine [55] make it particularly important for underserved communities [56].

### Cultural significance and empowerment through traditional medicine (FPEH Continued)

Beyond its economic function, traditional medicine holds profound cultural and spiritual significance, particularly for Cameroonian women. It is deeply embedded in local customs, traditions, and intergenerational knowledge transfer. As the study shows, many women practitioners view their work not only as healing but as a way to maintain cultural identity, preserve ancestral knowledge, and assert authority.

Traditional medicine thus becomes a site of cultural resistance—a means of reclaiming knowledge historically devalued by Western medical paradigms. This connects directly to the FPEH principles outlined in our framework, which contend that the subordination of women is not only economic but also epistemological. The marginalization of women’s knowledge systems, particularly in health practices, reflects wider patriarchal structures that suppress women’s voices and contributions. Traditional medicine therefore becomes a space for women to reclaim their power and challenge dominant, colonial health paradigms that have historically excluded or invalidated indigenous knowledge. As one participant articulated, this is about reclaiming cultural agency and resisting foreign healthcare systems that fail to acknowledge the cultural and historical dimensions of healing [57].

The study also sheds light on the systemic and institutional barriers women face in integrating traditional medicine into formal healthcare systems. Our findings, when viewed through the FPEH framework highlights how power relations within the health system—shaped by colonial legacies, patriarchy, and economic inequality—play a central role in excluding traditional medicine from official provision. Despite its widespread use, traditional medicine remains largely informal and unregulated, leaving women practitioners vulnerable to exploitation and marginalization. The lack of formal recognition, particularly for women, limits their participation in national health reforms. This reinforces gendered power imbalances, where women’s knowledge and practices are deemed inferior to Western biomedicine [20]. One participant conveyed her frustration that the combination of her gender and her lack of formal credentials led to her expertise being systematically dismissed and not taken seriously. This testimony vividly illustrates the epistemic injustice women face, where their knowledge is devalued for not aligning with dominant medical standards. Addressing these gendered and epistemological inequalities is key to decolonizing healthcare and making it more inclusive [27, 40].

This discussion links feminist political economy to both the cultural and economic significance of traditional medicine in Cameroon, emphasizing its role as both a source of empowerment and a crucial component of a more inclusive health system. Traditional medicine is vital in building inclusive health systems for Cameroonian women by offering accessible and affordable care, particularly where biomedical services are limited. Deeply rooted in cultural beliefs and community trust, traditional healers often provide the primary source of healthcare, making services culturally relevant and acceptable. Women are not only users of traditional medicine but also key knowledge holders and practitioners, contributing to their social and economic empowerment [27, 58]. It complements biomedical care by providing holistic and community-based support, especially in maternal health and emotional well-being. Its integration into national health policies supports the decolonization of healthcare systems and the promotion of gender-sensitive approaches. Furthermore, traditional practices play a crucial role in addressing mental and spiritual health needs often overlooked in clinical settings, thereby ensuring a more comprehensive approach to women’s health. Through intergenerational knowledge sharing, women healers help strengthen community resilience, ensuring vital health knowledge remains locally grounded and accessible.

### Ethical imperatives and a new era: implications of law No. 2024/018

Our findings must also be considered in light of the significant ethical challenges inherent in traditional medicine. As this research acknowledges, issues surrounding the lack of standardized practices, quality control in the preparation of remedies, and systematic documentation have long posed risks to patient safety and been a barrier to wider integration. The variability in diagnoses and treatments, while reflecting a rich diversity of knowledge, also creates an ethical imperative for oversight and regulation.

The recent enactment of Law No. 2024/018 marks a pivotal turning point in this regard [41]. This legislation, by establishing a formal system to ‘regulate and supervise’ the field, directly confronts these ethical concerns. It provides, for the first time, a legal pathway for developing standards, ensuring the quality and safety of remedies, and encouraging the documentation of practices. Therefore, this law can be interpreted as a foundational step in building the trust and accountability necessary to ethically merge traditional medicine into a more holistic national health system.

The enactment of Law No. 2024/018 is not just a contemporary administrative update; it signifies a significant shift in the historical trajectory of healthcare in Cameroon. This legislative formalization not only grants traditional medicine an unprecedented level of legitimacy but also acknowledges the pivotal role of its practitioners in the national health system, bridging the past and the future of healthcare in Cameroon.

This paves the way for a more comprehensive healthcare approach, potentially leading to improved health outcomes, especially in rural areas where traditional healers are often the primary care providers.

While the formalization of traditional medicine is a significant step, it also introduces new challenges.The regulatory process must strike a delicate balance, ensuring safety and efficacy without compromising the culturally embedded and often spiritual nature of traditional healing practices. The successful implementation of this law hinges on fostering a genuine partnership that respects the unique epistemologies of both medical systems.

As this paper has argued, this official recognition is the culmination of centuries of resilience, affirming that traditional medicine is not merely a healthcare modality but a living repository of Cameroonian cultural heritage.It is precisely this deep-rooted legacy that makes its practitioners indispensable. The new law not only provides the official framework to support and enhance their crucial role in extending primary healthcare to all Cameroonians but also underscores the value and respect for their contribution to the new healthcare system.

This law marks a critical step towards a uniquely Cameroonian health system that sustainably encompasses its rich medical heritage.

## Strengths and limitations of the study

### Strengths

This study offers a robust and nuanced examination of traditional medicine in Cameroon, grounded in several key strengths:

- First, its culturally grounded approach provides rich, context-specific insights by deeply engaging with the lived experiences of Cameroonian women who use and practice traditional medicine. This ensures the research respects and values indigenous knowledge without imposing external, Western-centric perspectives.
- Second, the explicit focus on gender and empowerment is central. By applying a Feminist Political Economy of Health framework, the study highlights women’s agency in healthcare, emphasizing their critical role in preserving and promoting traditional healing practices and centering their voices and authority within their communities.
- Third, the research provides a holistic understanding of health systems, moving beyond conventional biomedical views to explore the intersections of health, culture, and economy. This comprehensive perspective captures how traditional medicine addresses not only physical symptoms but also emotional, spiritual, and social well-being, offering a culturally sensitive model aligned with local needs.
- Fourth, the inclusion of diverse community-based perspectives from various regions across Cameroon broadens the study’s applicability and relevance. The use of direct participant quotes further authenticates the findings, lending credibility and depth to the analysis.
- Fifth, the study’s actionable policy recommendations are a significant strength. Developed through collaborative efforts, these recommendations aim to guide inclusive, gender-responsive healthcare reform, making the research valuable for stakeholders, government bodies, and NGOs seeking to integrate traditional medicine as a legitimate and complementary form of care.
- Finally, a core strength lies in its strong emphasis on the decolonization of healthcare. By critiquing the systemic marginalization of traditional medicine, the study challenges colonial legacies that continue to shape African health systems. Its call for integrating indigenous knowledge into national frameworks aligns with global decolonization movements, charting a path toward more equitable and culturally grounded healthcare futures.

In conclusion, this research’s strength lies in its cultural relevance, commitment to gender equity, and comprehensive view of health as both a social and medical phenomenon. By centering women’s experiences and advocating for the rightful place of traditional medicine in national health discourse, it significantly contributes to rethinking healthcare in Cameroon and beyond.

### Limitations

This qualitative study provides rich insights into Cameroonian women’s experiences with traditional medicine, yet it’s important to acknowledge its inherent limitations. The small sample size and non-random participant selection—driven by self-selection and the focus on specific regions (Centre, North West, South West)—limit the generalizability of findings across all Cameroonian women or other diverse contexts. This approach may overrepresent those deeply engaged with traditional practices, potentially underrepresenting skeptical perspectives or those relying more heavily on modern healthcare.

Additionally, a limitation of this study is the deliberate exclusion of high-level regulatory officials. While this was an ethical decision made to protect potential participants from professional risk, it means our findings do not capture the perspectives of those directly involved in enforcing current regulations on traditional medicine.

Furthermore, exploring sensitive health practices, especially those facing stigma, presented challenges in eliciting candid responses. Participants might have been hesitant to fully disclose their traditional medicine use, leading to incomplete data. The study’s multilingual context introduced potential language and translation issues, where the nuances of cultural meanings and idiomatic expressions might have been partially lost, impacting data richness.

Observational data of healing rituals was also limited by the private and dynamic nature of some practices, along with ethical and cultural sensitivities that restricted full engagement. Finally, while mitigation strategies like member checking and data triangulation were employed, researcher bias remains a possibility, as the researcher’s background and assumptions could have subtly influenced data collection and interpretation. The cross-sectional design also prevents a longitudinal analysis of how women’s engagement with traditional medicine evolves over time in response to changing societal and healthcare landscapes. Addressing these limitations in future research would significantly enhance our understanding of traditional medicine’s role in decolonizing healthcare for Cameroonian women.

## Recommendations

The findings of this study highlight the essential role traditional medicine plays in the healthcare of Cameroonian women, serving as a source of empowerment, cultural identity, and an accessible, holistic health resource. To leverage this value and foster inclusive, gender-sensitive, and decolonized health systems that honor women healers’ knowledge and practices, the following recommendations are crucial:

- *Formal and Equitable Integration:* The Cameroonian government should formally recognize and integrate traditional medicine into the national health system. This requires revising existing health policies to explicitly include traditional medical practices, with a strong focus on gender-sensitive policies that acknowledge and value the contributions of women healers. Crucially, certification programs for traditional healers should be developed to ensure official recognition and adherence to ethical and safety standards. Establishing collaborative frameworks between traditional and biomedical practitioners is vital for knowledge exchange and patient referrals, fostering a truly inclusive healthcare landscape.
- *Strengthen Education and Training for Women Healers:* Develop specialized training programs that blend ancestral knowledge with modern healthcare practices for women traditional healers. These programs should cover ethical standards, patient care, hygiene, and evidence-based techniques. Facilitate partnerships between universities, health institutions, and traditional healers to create accredited programs. Simultaneously, launch public health campaigns to promote the value of combining traditional and biomedical approaches for holistic care, and offer regular workshops to update healers on emerging tools and scientific developments.
- *Advocate for Gender-Responsive Policy Reform:* Incorporate gender-sensitive frameworks into health policies to directly address the systemic marginalization of women traditional healers. Policy reforms must promote the equal participation of women in healthcare decision-making, particularly in rural and traditional contexts. Partner with women's rights organizations to advance advocacy for the formal recognition and inclusion of women healers in national health strategies, complementing this with community-based awareness initiatives to legitimize women's roles in traditional medicine.
- *Promote Public Awareness and Acceptance:* Implement nationwide public health campaigns to educate the population about the benefits of traditional medicine, emphasizing its holistic and culturally grounded approach. Utilize diverse media channels (radio, television, social media) to disseminate information on how traditional healing complements biomedical practices. Engage influential community leaders and women’s health advocates to amplify these messages and organize community health fairs that bring together traditional and biomedical professionals to build trust and showcase integrated care models.
- *Address Socio-Economic Barriers to Access:* Improve access to traditional medicine for women in rural and marginalized communities by supporting local women healers to form cooperative networks or establish community clinics offering affordable care. Advocate for policies that regulate the costs of traditional treatments to ensure affordability across all economic groups. Encourage community-led initiatives that pool resources to enhance access, empowering women healers to serve populations often excluded from formal healthcare systems.
- *Prioritize Research on Traditional Medicine and Women’s Health:* Invest significantly in research focused on traditional medicine's impact on women’s health outcomes to inform policy and healthcare reforms. Foster partnerships among universities, health ministries, and traditional healers to conduct empirical studies evaluating the effectiveness of traditional practices and exploring their harmonization with biomedical care. Allocate dedicated research funding to explore the intersections between traditional healing, gender, and the decolonization of healthcare, ensuring findings are disseminated to policymakers and local communities for informed decision-making and practical implementation.

## Conclusion

This study underscores the unique benefits of traditional medicine for Cameroonian women, highlighting its deep cultural roots, accessibility, and empowering impact. Traditional healing, particularly women-led practices, offers holistic care and remains indispensable in rural and underserved areas, yet formal health systems often overlook these significant contributions. The profound impact of traditional medicine on women’s health is undeniable.

Traditional medicine, particularly when led by women healers, serves as a foundation for building more inclusive, culturally sensitive, and gender-responsive health systems in Cameroon. These healers, often deeply rooted in their communities, play a crucial role in fostering healthcare equity by addressing access gaps, empowering women, preserving indigenous knowledge, and promoting a holistic view of health that values both physical and spiritual well-being.

Our findings urgently call for the integration of traditional medicine into national healthcare policies, including providing training for women healers and enhancing their inclusion in decision-making processes. These reforms are crucial for establishing a more equitable, culturally responsive, and gender-sensitive healthcare system in Cameroon.

By valuing indigenous knowledge and addressing socio-economic barriers, Cameroon can make significant strides towards decolonizing healthcare. This approach not only ensures that all women, especially those from marginalized communities, receive the care and recognition they rightfully deserve but also presents a cost-effective solution to the country’s healthcare challenges.

This study opens the door for continued dialogue and collaborative efforts to explore the intersection of traditional medicine, gender, and healthcare reform. We invite policymakers, researchers, healthcare professionals, and practitioners to join us in this crucial conversation. Your expertise and commitment are vital in building inclusive health systems that honor Cameroon’s rich cultural heritage and ensure equitable access to healthcare for all women.

## Supplementary Information

Supplementary Material 1.

## Data Availability

The datasets generated and analyzed during the current study are not publicly available due to privacy and confidentiality agreements with participants but are available from the corresponding author on reasonable request, subject to ethical approvals.

## Declarations

The authors confirm that all research involving human participants and human data was conducted in accordance with the ethical principles of the Declaration of Helsinki.
