# Supplementary material for: Decolonizing healthcare: the role of traditional medicine in building inclusive health systems for Cameroonian women
Source: BMC Complement Med Ther. 2025 Oct 17;25:384. doi: 10.1186/s12906-025-05133-0 (PMC12535010; doi:10.1186/s12906-025-05133-0)
Supplement: Supplementary file 1 — Supplementary Material 1. [file 12906_2025_5133_MOESM1_ESM.docx]

**Interview Guide**

**Title**: *Decolonizing Healthcare: The Role of Traditional Medicine in Building Inclusive Health Systems for Cameroonian Women*

Good morning/afternoon. Thank you for agreeing to participate in this interview. My name is XXX, and I am conducting a study on the role of traditional medicine in Cameroon—especially how it is experienced, practiced, and understood by women. The purpose of this study is to understand and document the unique perspectives and experiences of Cameroonian women regarding traditional medicine and how this knowledge can inform the development of more inclusive and culturally responsive healthcare systems. I am interested in how you define and relate to traditional medicine, and your insights will be invaluable in shaping the future of healthcare in Cameroon.

I would appreciate hearing about your experiences, reflections, and ideas. There are no right or wrong answers—what matters most is your honest perspective. Everything you share will remain confidential and anonymized in the research. This means that your name and any identifying information will be replaced with a code, and only I will have access to the key that links the code to your identity. You are free to skip any question or stop the interview at any point.

With your permission, I would like to record the interview to make sure I accurately capture your words. May I proceed with the recording?

*(Tip to interviewer: Use gentle probes as needed, such as: “Could you tell me more about that?”, “Can you give an example?”, “How did that feel for you?”, “Why do you think that is?”)*

**A. Background and Context**

1. Could you please tell me a little about yourself (e.g., age, community, profession, or role)?
2. What is your relationship or experience with traditional medicine?
3. How would you describe traditional medicine as it is practiced or understood in your community?

**B. Lived Experiences and Cultural Significance**

1. How did you come to use or practice traditional medicine?
2. What does traditional medicine mean to you personally—as a woman, a healer, or a member of your community?
3. What kinds of health conditions or needs do women in your community usually address through traditional medicine?
4. Are there particular rituals, plants, or healing methods that are especially important or symbolic for women in your community?
5. How is knowledge of traditional medicine passed on—especially among women and across generations?
6. Do you feel that traditional medicine contributes to women's empowerment in your community? If so, how?

**C. Social and Economic Value**

*(For healers)*

10. Does practicing traditional medicine contribute to your household income or financial independence?

*(For non-healers)*

10. How does relying on traditional medicine (compared to using formal healthcare) affect your household finances or economic independence?

1. How are women who are traditional healers perceived or treated in your community?
2. In your opinion, what is the overall status or value given to traditional medicine and those who practice it today?

**D. Institutional and Policy Barriers**

1. How do you view the relationship between traditional medicine and the national (biomedical) healthcare system in Cameroon?
2. Have you had any interactions or collaborations with hospitals, clinics, or biomedical professionals? What was that experience like?
3. Do you feel that traditional healers are respected or recognized by health authorities or policymakers?
4. Are there any rules, policies, or challenges that make it difficult to practice or use traditional medicine—especially as a woman?
5. What kind of support or recognition would help you strengthen your work or improve your access to traditional medicine?

**E. Building Inclusive and Decolonized Health Systems**

1. In your view, what would an inclusive and culturally respectful healthcare system look like?
2. What role do you think traditional medicine—especially women-led practices—can play in improving health in Cameroon?
3. What recommendations would you give to the government or health authorities for better integration of traditional medicine into the national healthcare system?
4. How can we ensure that women's voices and knowledge are included in national health decision-making and policy development?

**F. Final Reflections**

1. Is there anything else you would like to share that is important and has not been covered?
2. Do you have any questions for me about the study or how the information will be used?

Thank you so much for your time and for sharing your experiences. Your voice is important and will help us better understand how traditional medicine can be recognized and integrated into the health system in a way that respects women's knowledge, choices, and rights. Your insights could potentially influence healthcare policies and practices in Cameroon. If you would like, I can share the study's findings with you once it is complete. Please let me know if that interests you. Wishing you all the best.
